# Influence of Membrane Phase on the Optical Properties of DPH

**DOI:** 10.3390/molecules25184264

**Published:** 2020-09-17

**Authors:** Silvio Osella, Markéta Paloncýová, Maryam Sahi, Stefan Knippenberg

**Affiliations:** 1 Chemical and Biological Systems Simulation Lab, Centre of New Technologies, University of Warsaw, Banacha 2c, 02-097 Warsaw, Poland; 2 Regional Centre of Advanced Technologies and Materials, Department of Physical Chemistry, Faculty of Science, Palacký University Olomouc, 17. listopadu 12, 771 46 Olomouc, Czech Republic; marketa.paloncyova@gmail.com; 3 Department of Theoretical Chemistry and Biology, School of Engineering Sciences in Chemistry, Biotechnology and Health, KTH Royal Institute of Technology, SE-10691 Stockholm, Sweden; msahi@kth.se; 4 Theory Lab, Hasselt University, Agoralaan Building D, 3590 Diepenbeek, Belgium

**Keywords:** conformationally versatile molecules, QM/MM, absorption, photoselection, fluorescence decay, fluorescence anisotropy, hyper-Rayleigh scattering, two-photon absorption

## Abstract

The fluorescent molecule diphenylhexatriene (DPH) has been often used in combination with fluorescence anisotropy measurements, yet little is known regarding the non-linear optical properties. In the current work, we focus on them and extend the application to fluorescence, while paying attention to the conformational versatility of DPH when it is embedded in different membrane phases. Extensive hybrid quantum mechanics/molecular mechanics calculations were performed to investigate the influence of the phase- and temperature-dependent lipid environment on the probe. Already, the transition dipole moments and one-photon absorption spectra obtained in the liquid ordered mixture of sphingomyelin (SM)-cholesterol (Chol) (2:1) differ largely from the ones calculated in the liquid disordered DOPC and solid gel DPPC membranes. Throughout the work, the molecular conformation in SM:Chol is found to differ from the other environments. The two-photon absorption spectra and the ones obtained by hyper-Rayleigh scattering depend strongly on the environment. Finally, a stringent comparison of the fluorescence anisotropy decay and the fluorescence lifetime confirm the use of DPH to gain information upon the surrounding lipids and lipid phases. DPH might thus open the possibility to detect and analyze different biological environments based on its absorption and emission properties.

## 1. Introduction

Lipid membranes are known to be complex systems which exhibit a plurality of vital functions, as they both protect the cell from outer environments and play an active role in the transport of ions, nutrients or even drugs within the cell. In addition, they also provide sub compartmentalization and molecular organization of critical cellular processes. Structurally, lipids can form planar lipid bilayers, while other arrangements such as monolayers or hexagonal structures can be found in organisms present, for example, in mammalian eyes and lungs or in fusing vesicles, respectively [1,2,3,4]. Yet, in cellular membranes of both prokaryotic and eukaryotic cells, lipid bilayer structures are dominantly present.

To maintain their biological functions, membranes need to possess a high degree of fluidity. This parameter is directly related to the temperature and the composition of the membrane itself, and can vary depending on the nature of the fatty acid molecules composing the membrane but also on the presence of different components, such as sphingomyelin and cholesterol. It has been indicated in the literature that there is a direct connection between the fluidity of the membrane and the rising of different illnesses [5,6,7,8,9]. In particular, increased levels of cholesterol decrease the membrane fluidity and alter the spatial organization of membrane nano- and micro-domains and nourish the hypothesis around the existence of so-called lipid rafts [10,11]. The presence of different domains in membranes manifests itself through their changing softness, and is a direct result of varying degrees of fluidity; this heterogeneity may be of interest in the dissemination of metastatic tumors [12]. In fact, fluidity indicates how molecules move within the membrane, as well as the rate of this movement, and is inversely related to the microviscosity. Thus, knowing the characteristics of membrane fluidity is paramount to the understanding of complex mechanisms which are regulated by the membrane properties [13]. 

It has been suggested in the literature that there is a direct link between membrane fluidity and the presence of pathologies, including chronic inflammatory conditions, carcinogenesis, cardiovascular diseases, and even aging. Changes in membrane fluidity of tumor cells affect antigens and receptors [6,14] and cell motility [15], as well as the capacity of deforming potential cancer cells [16]. Cancer cells lead to the alteration of many biochemical processes, including the lipid metabolism. Thus, differentiating between the nature of malignant and non-malignant cells is an urgent, yet not easy task. The composition of lipids in membranes is strongly dependent on the type of cancer, leading to a strong variability [17,18]. Moreover, in the progression of the tumor, cancer cells present specific alteration of lipid synthesis, leading to a different lipid composition of the same tumor depending on its stage. It is thus of high importance to be able to detect changes in membrane fluidity in order to assess the healthiness of cells. 

In recent years, different experimental techniques have been used for this purpose, and among them, fluorescence techniques and other imaging spectroscopy techniques have been demonstrated to be able to determine that both microscopic and nanoscopic membrane domains contribute to the function of living organisms, and have assessed the different fluidity of these domains [1,19,20,21,22,23,24,25,26]. However, the details of nanoscopic membrane organization in cells remain elusive and strongly understudied. An alternative approach is therefore needed to investigate the mechanisms regulating the nanoscopic organization of lipid membranes. One option is to use model lipid membranes, such as multi- or unilamellar vesicles and supported planar bilayers, to study membrane organization in vitro. A second option is to rely on computation for modelling and predict the properties of lipid bilayers.

Computation has become more and more reliable and able to unravel the properties of complex environments [27], thanks to a multiscale approach, which allows to study large portions of model membranes with classical molecular dynamics (MD) simulations. Afterwards, the focus can be put on a smaller part of the system to study optical properties by means of hybrid quantum mechanics/molecular mechanics (QM/MM) methods. In this way, many different properties such as linear and non-linear optical properties, fluorescence and photoselection of an optical probe immersed into the membrane can be assessed [28,29,30,31,32,33,34]. The ability of the probe to give different optical responses depending on the environment it is embedded in allows to determine the phase of the membrane itself, which, in turn, is strongly related to its fluidity.

In this paper, we study the optical properties of a well-known probe, diphenylhexatriene (DPH), embedded in different membrane phases, by means of a well-established QM/MM approach [29,30,31,32,33]. Model membranes have been considered, consisting of dioleoylphosphatidylcholine (DOPC), dipalmitoylphosphatidylcholine (DPPC) and a 2:1 mixture of stearoyl sphingomyelin (SM) and cholesterol (Chol) for the liquid-disordered (Ld), solid-ordered (So) and liquid-ordered (Lo) phases, respectively. We show that by combining different optical analyses, it is possible to screen between the different membrane phases using the same DPH probe. 

## 2. Results and Discussion

### 2.1. QM and MD Dihedral Analyses

**Figure 1:**
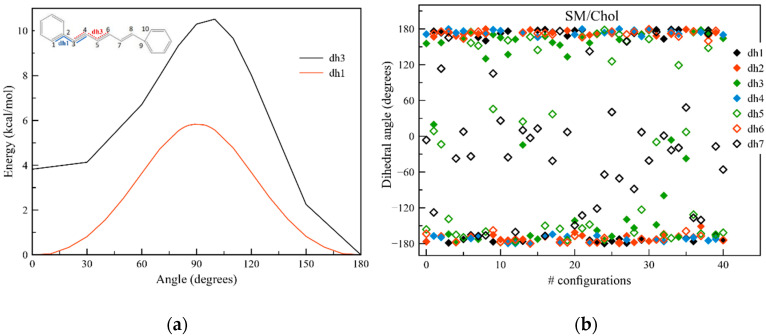
(**a**) Dihedral angle energy barriers for dh1 (1-2-3-4) and dh3 (3-4-5-6) of DPH (diphenylhexatriene). All values have been obtained in vacuum, at the B3LYP/cc-pVDZ level of theory. The inset shows the ttt isomer of DPH; the numbers refer to the atoms used for the dihedral analysis; (**b**) Values of the 7 dihedral angles of DPH observed in all 40 snapshots selected over the trajectories for SM/Chol (Lo). dh7 is defined with respect to the opposite side of the phenyl ring compared to dh1.

**Figure A1:**
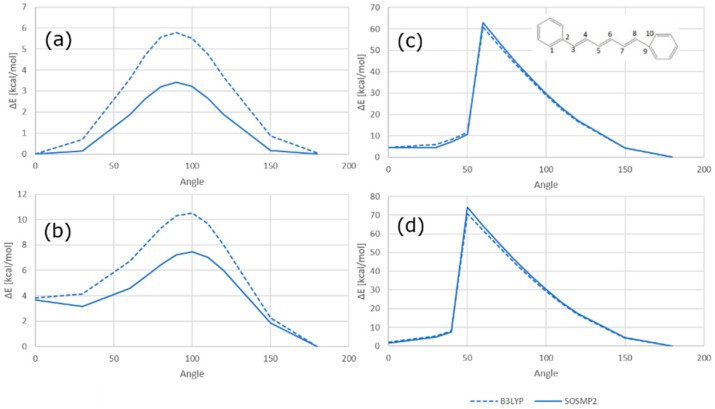
Ground state rotational barriers for (**a**) dh1 (1-2-3-4), (**b**) dh3 (3-4-5-6), (**c**) dh2 (2-3-4-5) and (**d**) dh4 (4-5-6-7) of DPH at the B3LYP/cc-pVDZ and SOSMP2/cc-pVDZ level.

To get information about the ground state properties of the molecule and the conformational versatility of the DPH probe, the rotation barriers in vacuum were calculated. In Figure 1, the energetical differences between the rotation over the dihedral angles dh1 and dh3 are depicted, while the other dihedral angles are considered in the trans conformation. It can be noted that we only consider the rotation over the single bonds, since the double bond rotations are energetically forbidden with barriers reaching 70 kcal/mol (within the framework of the methodology used, see Figure A1 in Appendix A). The energy differences between the maxima of dh1 and dh3 and the global minimum of the all trans conformation amount to 5.9 kcal/mol and 10.2 kcal/mol, respectively (Figure 1). Thus, the flexibility of DPH will be strongly characterized by the rotation of the phenyl groups. A benchmark of these values has been made, too: as B3LYP tends to favor planar structures which generally results in too high barriers and large dihedral angles (due to the underestimation of dispersion forces [35,36]) and on the contrary, since MP2 results into too compact compounds with decreased inertia moments [37], we reverted to SOS-MP2 for the energetics of the dihedral angles and obtained values of 3.3 kcal/mol and 7.8 kcal/mol for dh1 and dh3, respectively, confirming the trend from the DFT results (Figure A1). Both B3LYP and SOS-MP2 report an energy difference of 4 kcal/mol between the cis and the more stable trans conformation for dh3. 

The high rotation barriers over the double bonds lead to the presence of eight different conformers, of which only six are energetically different based on the methods used in the current study [38]. According to Boltzmann statistics at ambient temperatures, the ttt conformer is the most abundant one (94%), while the remaining part is mainly due to the tct conformer (see discussion in Appendix A). In this work, we focus on the most abundant all trans conformer and investigate whether the conformational versatility due to the rotations over the single bonds can be used to study the phase of the surrounding lipid bilayer by means of (non) linear optical spectroscopies and fluorescence.

**Figure A5:**
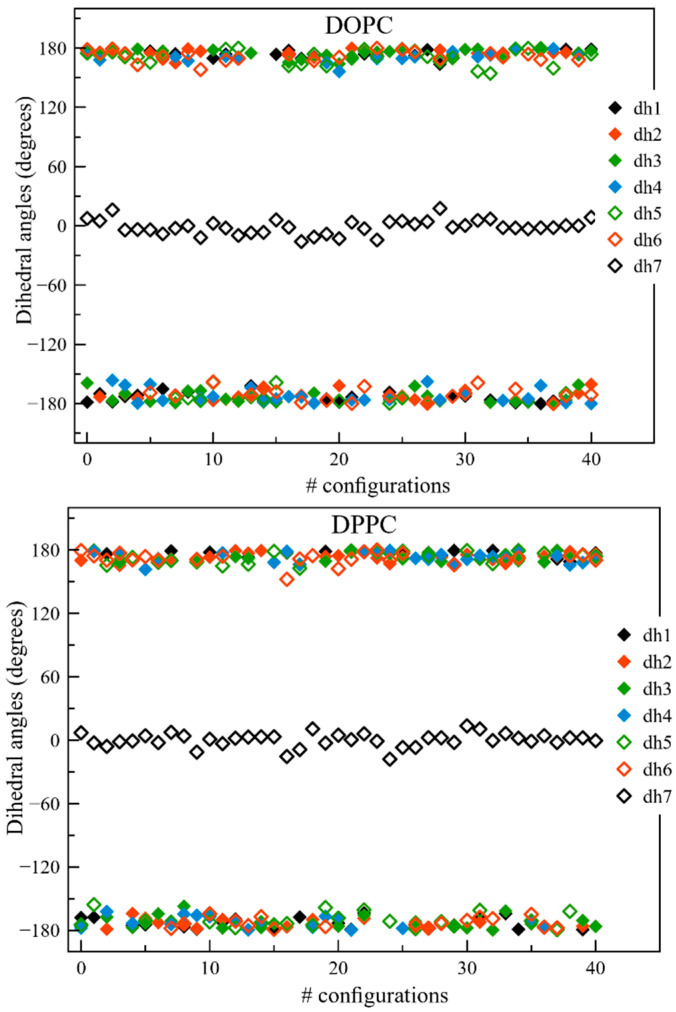
Values of the 7 dihedral angles of DPH observed in all 40 snapshots selected over the trajectories for DOPC (Ld) and DPPC (So). dh7 is defined with respect to the opposite side of the phenyl ring compared to dh1.

Although the ambient temperatures applied to the lipid membranes are not sufficient to overcome these barriers, the steric hindrance and electrostatic influence of the environment might influence the conformation of DPH in the three phases. Therefore, from the 500-ns-long classical molecular dynamics (MD) simulations performed in our earlier study [39], 40 frames were extracted in the last 200 ns for each membrane phase. For each snapshot the seven dihedral angles were analyzed. In line with the discussion above, dh1 and dh7 involve the dihedral angle of both phenyl rings over the first single bond. Yet, dh7 is defined with respect to the opposite carbon atom of the phenyl group. As a consequence, in both DOPC (Ld) and DPPC (So) dh1 is solely found in a trans conformation, while dh7 is consistently found in a cis conformation (Figure A5), and the all trans conformation of DPH is preserved during the MD simulation. For SM/Chol (Lo) however, dh7, dh5 and to a lesser extent dh3 in the middle of the chain do change their value tremendously as time propagates (Figure 1b). As a result, these changes influence the magnitude and orientation of the transition dipole moment of DPH with respect to the z-axis. This means the photoselection of the membrane probe will be heavily affected by the conformation. Since the different orientations will result in different influences of the anisotropic environment, these conformational changes may be visible in the optical spectroscopies as well. From the thorough investigation of the position of the probe in the three membranes performed in our previous study [39], we know that the center of mass of the probe increases the distances from the membrane center going from DPPC (So) over DOPC (Ld) to SM/Chol (Lo). Furthermore, in view of the rather vertical orientation of the probe in the respective membranes, it is clear that one of the two phenyl rings is closer to the polar groups and the water phase while the other one is buried into the aliphatic phase. For SM/Chol, this proximity to the membrane surface is enough to force changes in the outermost dihedral angles, while gradually those dihedral angles along the backbone of DPH closer to the membrane center stay indifferent to the membrane specificities. In contrast to DOPC and DPPC with the observed rather rigid molecular configurations, DPH is flexible in SM/Chol and comes in close contact with polarized groups. 

### 2.2. One Photon Absorption

**Figure 2:**
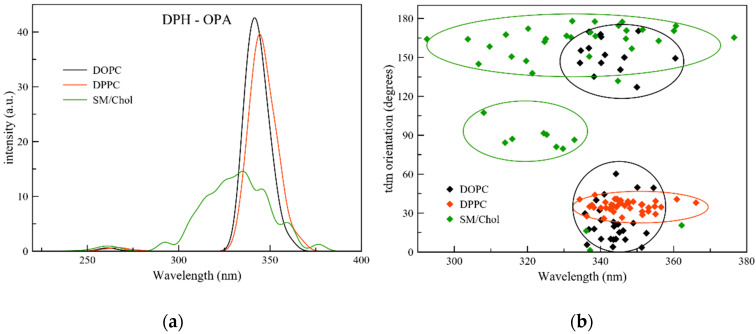
(**a**) One photon absorption spectra of DPH in the three different membranes; (**b**) Correlation between absorption wavelength and orientation of the transition dipole moment (tdm) of the extracted snapshots of DPH in the different membranes.

The above selected snapshots were used in the QM/MM approach and calculations of the optical properties were performed. The one photon absorption (OPA) spectra of DPH in the different membranes is reported in Figure 2.

**Figure A6:**
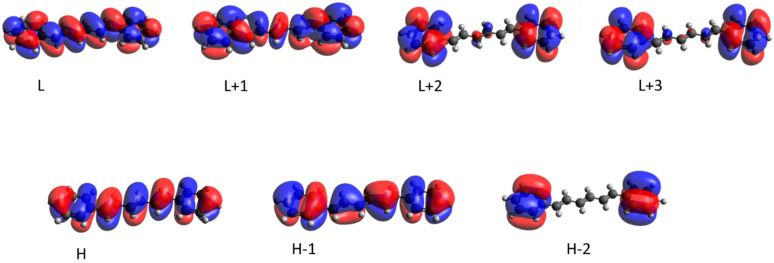
Frontier orbitals of DPH at the CAMB3LYP/cc-pVDZ level of theory. H and L denote the highest occupied and lowest unoccupied molecular orbital, respectively.

The lowest lying excited state S1 is the only active one for the absorption, with a strong oscillator strength value, while S2 and S3 are virtually dark states. In all different membranes the S1 absorption of DPH relates to a transition from the HOMO to the LUMO orbital (see Figure A6) at wavelengths of 336 nm, 346 nm and 337 nm for DOPC, DPPC and SM/Chol, respectively. In addition, the lambda overlap parameter of Peach et al. [40] confirms that in all membranes this transition has a local character (Table A2). The absorption spectra in DOPC and DPPC are similar with a slight red shift of 10 nm for the latter. From a previous analysis of the DOPC and DPPC membranes, we know that water molecules can be found up to 1.3 nm and 0.9 nm from the membrane center [41], respectively, which might corroborate this red shift. Rather big differences are observed when the probe is immersed in the SM/Chol mixture. In this last membrane, the absorption is much broader than in DOPC and DPPC, but still related to the S1 state. The analysis of the orientation of the transition dipole moment (tdm) of DPH in the extracted frames explains the behavior obtained. In DPPC a single orientation of 36° is found, which can explain the single peaked spectrum obtained. In DOPC two different tdm distributions are present, with orientations in the 20–60° window and at higher values in between 120 and 160°. In light of the symmetry of DPH, these two minima denote the same orientation. Since the tdm orientation is considered as the angle between the tdm vector and the normal to the membrane, these two minima are also a manifestation of the so-called flip-flops which has been reported previously for DOPC [39]. As a consequence, both distributions fall in the same wavelength zone, thus explaining the single peaked spectrum obtained. A different situation arises when the probe is immersed in SM/Chol. Now, the tdm orientation has a value of 160° on average (with a second, smaller population around 90°) but is spread over a vast variety of wavelengths. This relates to the conformational changes which are amply present when the probe is embedded in this lipid bilayer as discussed above. The second population of DPH molecules embedded in SM/Chol (8 frames out of 40) has a transition dipole moment which is oriented perpendicular to the z-axis of the membrane. The differences in localization of the probe in the different membranes are here important, too, with a position much closer to the membrane surface when DPH is immersed in the SM/Chol mixture [39]. Using the analysis in [41], we find that the density around the probe is higher in the case of SM/Chol (~ 950 kg/m^3^) than for DPPC (~850 kg/m^3^), which results in a higher influence of the environment on the optical spectra through steric hindrances and close interactions with polar groups. Thus, the different depths of the probe, and the relative orientation of DPH to the membrane normal, lead to strong differences in OPA for the Lo phase. 

When an impinging light beam with field vector parallel to the z-axis of the lipid bilayer is considered, the efficiency of the photoselection diminishes as the angle of the transition dipole moment with the z-axis increases. Consequences of this can be seen in the spread of the orientation of the tdm displayed in Figure 2, suggesting that DPH can screen between the three phases presented here, leading to a strong difference in absorption spectral shape when embedded in the Lo phase.

### 2.3. Non-Linear Optical Properties

Different non-linear optical (NLO) properties are presented to assess the ability of DPH to screen between the different membrane phases. In particular, we focus on two photon absorption (TPA) and hyper-Rayleigh scattering (HRS), which are promising experimental spectroscopies for biomedical applications. As such the outcome of these spectroscopies is largely determined by the excited state and transition state dipole moments between excited states. 

#### 2.3.1. Two Photon Absorption

**Figure 3:**
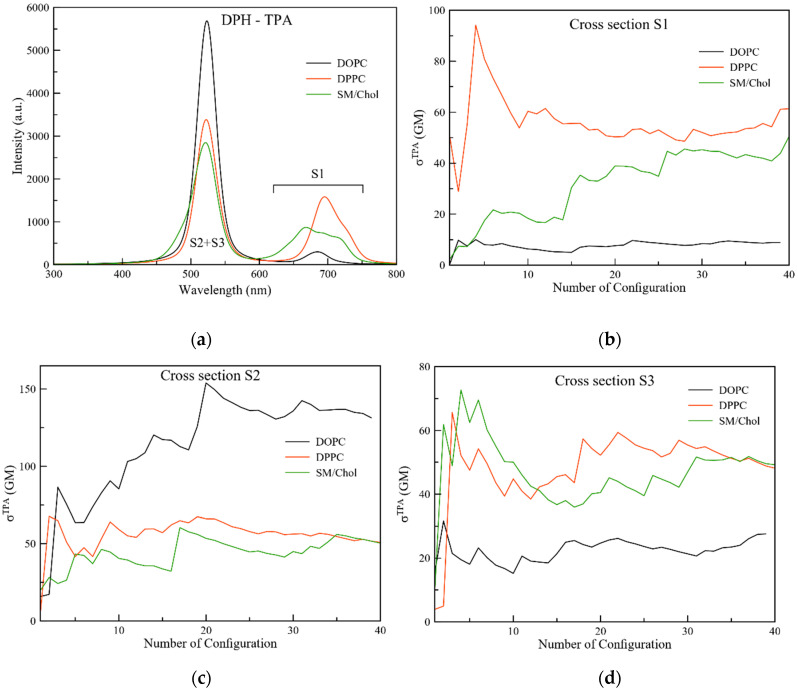
(**a**) Two photon absorption spectra of DPH in the three different membranes; (**b-d**) Cross section of the low-lying excited states of DPH in the different environments.

TPA is the method of choice when considering the use of probes in living organisms, since it requires laser beams at red or infrared light frequency, causing less damage to the surrounding tissues and cells. Moreover, it has a higher penetration depth with respect to OPA and it enables 3D imaging. From our computations we observe a TPA in the infrared part of the spectrum, with the S1 state lying at 687, 693 and 665 nm when DPH is in DOPC, DPPC and SM/Chol, respectively. On the other hand, a similar absorption is found for both S2 and S3, in the 620–630 nm range, but the intensities are strongly dependent on the membrane phase (Figure 3).

When the cross section is analyzed for S1, differences arise depending on the nature of the membrane; in particular, for this excited state DPPC is the active phase, with a value of 60 GM followed by SM/Chol (50 GM), while in DOPC the S1 state is dark (low value of 9 GM). On the other hand, DOPC shows the dominant contribution when S2 is considered, with cross section values up to 130 GM, while a similar value of 50 GM is found in both DPPC and SM/Chol. Remarkably, while there is a pretty large variety of cross section values in the DOPC membrane depending on the excited state considered, similar values for all three excited states are observed for DPPC and SM/Chol. The standard deviations for the displayed TPA values are given in Table A3. It can be remarked that they do not exceed 30% for all three states in the different probes. Attention can be paid to the 8 frames in the SM/Chol case with the DPH transition dipole moment perpendicular to the z-axis (frame numbers 5–12 in the depictions of the TPA cross sections in Figure 3). For the S1, a first plateau of cross section values of around 20 GM is obtained, which then increases for the remaining frames which are now oriented parallel to the membrane tails. For the S3, however, the cross section values of this second population are vastly higher than for the other ones. It can be remarked that these calculations have been performed in the absence of an explicit membrane potential. Since cholesterol is known to influence or rather enforce it [42,43], we foresee that the TPA cross section values might be affected, and the differences for the SM/Chol membrane with respect to DOPC and DPPC might increase, too. 

#### 2.3.2. Hyper-Rayleigh Scattering

The hyper-Rayleigh scattering (HRS) analysis allows us to investigate molecules which do not have a ground state dipole moment. In contrast to the coherent electric field induced second harmonic generation (EFISHG) technique, which also bears the first hyperpolarizability β, no external electric field has to be applied. Since HRS can be applied to ionic and octopolar molecules and can be combined with electrochemistry to probe structural changes by oxidation and reduction, this technique is nowadays one of the most used. It can be remarked that the DPH probe loses its centrosymmetry when embedded in a biological environment. We focus here both on the static and the dynamic hyperpolarizabilities. In the former, the frequency of the impinging field is theoretically set to 0 to assess the intrinsic contribution of the probe. For the latter, an impinging signal of 810 nm has been considered, which allows for a high light transmission in case of fluorescence probes [44,45], collagen [46], cellulose and plant polysaccharides [47], and second harmonic generating nanoparticles [48] and is therefore nowadays commonly used in experimental set ups. The wavelength chosen here can be found in the middle of the so-called therapeutic window (690–1040 nm), avoiding absorption by water which may otherwise cause unwanted heating effects [49]. 

**Figure 4:**
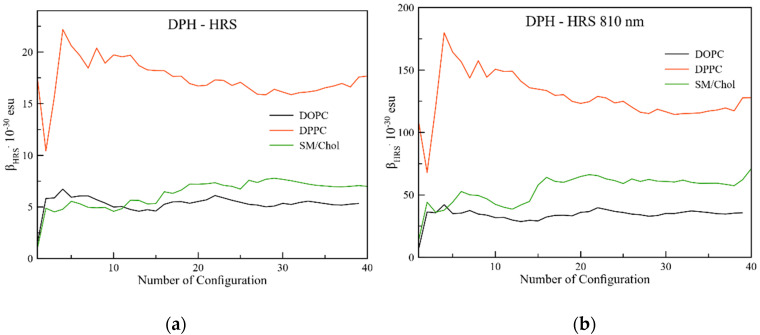
(**a**) Static and (**b**) dynamic components of the hyper-Rayleigh scattering.

The analysis is reported in Figure 4a. As already observed for TPA, DPPC is also the active phase for HRS, with values up to 18 × 10^−30^ esu while similar values of 7 and 5 × 10^−30^ esu were obtained in SM/Chol and DOPC, respectively. For a discussion on the standard deviations of these values, we refer to Appendix C and Table A4. Incorporating an impinging near infrared laser beam (Figure 4b), the intensities are ten times enhanced but the relative trend among the different lipid bilayers does not change. Thus, the current work enforces the ample studies performed in the static regime; incorporation of a well-tuned light beam might be opportune to mimic specific experimental conditions [50] or particular complex probes [34], but in the context of the current studies it does not incorporate new physics. It can therefore be stated that the static hyperpolarizabilities have a profound universal character.

As a conclusion on the analysis of the non-linear optical properties, both TPA (S1) and HRS analyses confirm that DPH is a good probe for the So phase recognition. To discriminate the Ld membrane phase from the Lo and So ones, the TPA cross section of the S2 excited state of DPH can be used.

### 2.4. Fluorescence Properties

Fluorescence spectroscopies are nowadays one of the techniques of choice for the analysis of the behavior of probes in different membrane phases, due to the different responses related to the anisotropy decay [51,52,53,54,55,56,57]. We focus on the decay time and the fluorescence anisotropy decay as model analyses which can be obtained by computations [29,30,31,33,34]. The decay time analysis, which can be closely related to the fluorescence lifetime imaging (FLIM) technique, allows screening between the membrane phase and gives indication on the phase itself.

**Figure 5:**
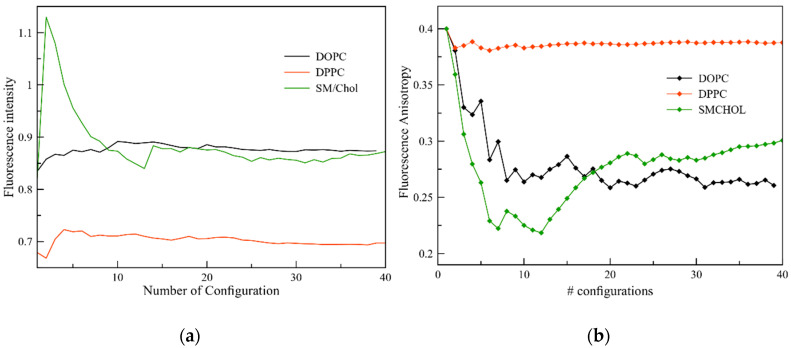
(**a**) Cumulative average of the fluorescence decay time; (**b**) Fluorescence anisotropy decay.

Figure 5a reports the fluorescence lifetime of the extracted frames of DPH in the different membranes, calculated by means of Einstein’s coefficient for spontaneous emission, Equation (3). The averaged decay curves for DPH embedded in DOPC and SM/Chol report a lifetime of ~0.87 ns, while the one for DPPC is slightly shorter (~0.69 ns). Once more, this analysis confirms the ability of DPH to recognize the solid gel phase among the three proposed here. 

To move a step further, we consider the fluorescence anisotropy analysis, which is directly related to the ease of rotation of the probe in the anisotropic membrane environment. In fact, the more the rotation is hindered, the stronger the anisotropy is maintained, and its decay suppressed. In lipid membranes where the rotation is allowed, the opposite is however true. As shown in Figure 5b, very different anisotropy decays are obtained depending on the phase of the membrane in which DPH is embedded. In particular, a negligible decay (to 0.38) is found in DPPC, as expected due to the strong hindrance in rotation. On the other hand, the fluorescence anisotropy decays rather profoundly for the DOPC membrane; from frame 20 on, a limiting value of 0.22 is obtained. A different behavior is obtained when DPH is embedded in the SM/Chol mixture; for the first few configurations the anisotropy decays steeply due to the half flip of the DPH molecule and the perpendicular position of the probe with respect to the z-axis of the membrane. From frame 13 on, the anisotropy raises again as the DPH probe returns to its orientation rather parallel to the z-axis, reaching a final value of 0.3, in between the other two phases. 

**Figure 6:**
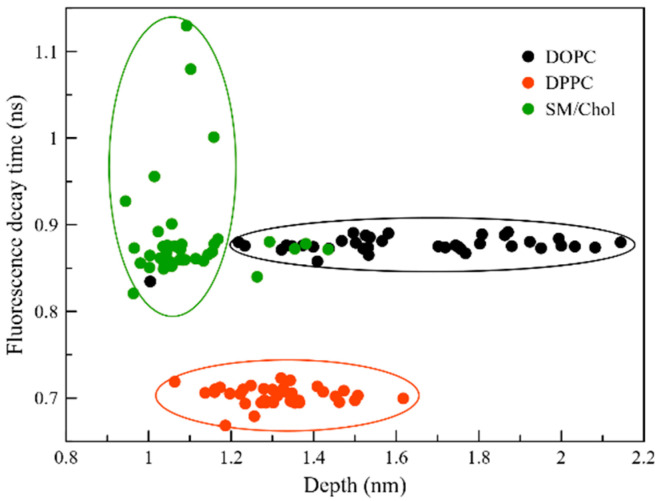
Correlation between location of the DPH probe in the membrane and the fluorescence decay time. The depth is defined as the difference between the phosphorous atoms at the membrane surface and the center of mass of DPH.

To rationalize these results, we performed an additional analysis considering the relation between the decay time and the localization of DPH in the different membranes (Figure 6). Once more, differences are present when considering DOPC and DPPC versus SM/Chol. In particular, for both Ld and So phases the analysis shows a large spread in the location of the probe, going from 1.1–1.6 nm window in DPPC to 1.3–2.1 nm range in DOPC, while the spread in fluorescence decay time is very limited. The opposite is observed in the SM/Chol mixture; now the depth of the probe has a very small spread over the average value of 1.1 nm, but the decay time presents a large spread in value, from 0.7 up to 1.2 ns. Thus, if we recall the above analysis of the decay times, we can now assert that in DOPC and SM/Chol the decay time is similar but for opposite reasons.

## 3. Materials and Methods 

To get insight into the conformational changes of the DPH molecule, the different conformers were optimized both at the B3LYP and the Scaled Opposite Spin Møller Plesset (SOS-MP2) level of theory along with Dunning’s cc-pVDZ basis set [58] and the Q-Chem package of programs [59]. The reference dihedral angle was changed in steps of 30°. 

From the 500–700 ns long molecular dynamics (MD) calculations performed in our previous study on the orientational distribution of DPH in lipid membranes [39], 40 snapshots were randomly selected out of the NPT production runs for DOPC, DPPC and a 2:1 mixture of sphingomyelin and cholesterol (SM/Chol). Since for these three membranes the temperature was maintained at 298K, three different lipid phases were obtained: liquid disordered phase (Ld) for DOPC, solid gel (So) for DPPC and liquid ordered (Lo) for SM/Chol. For all three membranes, a cutoff of 2 nm has been applied around the membrane molecules surrounding the probe. The long axis of the cylinder was oriented parallel to the normal to the membrane. A semi-spherical cutoff of 1.5 nm was used for the water layer in close proximity to the membrane. Since the properties of the simulated membrane phases have been described and validated in our previous studies, using the same computational methodology [39,41,60], we restrict ourselves here to a concise overview. For each phase, one fluorophore has been inserted into a membrane of 64 lipids per leaflet surrounded by 4500 water molecules described by the extended single point charge (SPC/E) model. To the bulk water, Na^+^ and Cl^-^ ions were added to a 0.9% physiological concentration. 

In the electrostatic hybrid quantum mechanics/molecular mechanics (QM/MM) approach, the obtained systems are split into two parts: the DPH probe itself whose linear and non-linear optical properties were calculated by means of time-dependent density functional theory (TDDFT), the CAM-B3LYP long range corrected functional [61] and the cc-pVDZ basis set, and the membrane lipids, ions and solvent molecules which have been described using the Gromos 43a1-s3 force field charges [62,63,64]. Hydrogen atoms were added by adjusting the pH level [65,66]. TDDFT calculations were performed using the Dalton2016 program [67,68]. The combination of the functional and basis set used in the current study has been benchmarked against other density functionals and post-Hartree Fock methods in our previous studies [29,30,31,32]. 

We focus here on one-photon absorption (OPA), two-photon absorption (TPA), hyper-Rayleigh scattering (HRS) as well as fluorescence spectroscopies. For OPA, an excited state *e* with energy *ω* and transition dipole moment *μ_eg_* from the ground state is characterized by an oscillator strength [69]

(1) $$f=\frac{2}{3}\omega \mu_{eg}^{2}$$

The TPA cross section in Göppert-Mayer units (GM) is obtained by

(2) $$\sigma {\left(\omega \right)}_{GM}=\frac{8\pi^{2}\alpha^{2}a_{0}^{4}t_{0}}{\Gamma }\delta \left(\omega \right){\left(\frac{\omega }{2}\right)}^{2}$$

with *α* the fine structure constant, *a*_0_ the Bohr radius, *t*_0_ the atomic unit for time, *Γ* the Lorentzian broadening of 0.1 eV, *ω* the OPA excitation energy and *δ* the TPA strength in au [70]. 

The HRS signal is measured by unpolarized optical excitation and can be calculated as $\langle \beta_{HRS}\rangle =\sqrt{\langle \beta_{ZZZ}^{2}\rangle +\langle \beta_{XZZ}^{2}\rangle }\;$. The capital letters denote lab frame coordinates, while brackets represent the orientational distribution average of the molecule in the environment. In the frame of the molecular coordinates, these averages can be expressed as a combination of *β_ijk_* tensor components. The equations are fully discussed in references [31] and [71]. 

The radiative lifetime *t*_0_ can be calculated making use of the rate *Γ*_0_ for spontaneous emission as *τ_0_*= 1/*Γ*_0_ and

(3) $$\Gamma_{0}=\frac{4}{3}\frac{{\left|\mu_{eg}\right|}^{2}}{4\pi \varepsilon_{0}\hbar }{\left(\frac{\omega_{eg}}{c}\right)}^{3}$$

where $\mu_{eg}$ and $\omega_{eg}$ are respectively the transition dipole moment and the transition frequency for the first one-photon allowed excited state *e* of the probe relative to the ground state *g*; $\varepsilon_{0}$ denotes the permittivity of vacuum, ℏ the reduced Planck constant and *c* the light speed constant [72]. In a solvent or biological environment, the change of the dielectric properties has to be taken into account. Therefore, the energy of the emitted photon is renormalized through $\varepsilon_{0}\to \varepsilon_{r}\varepsilon_{0}$ and c→c/n substitutions, with $\varepsilon_{r}$ the relative permittivity and *n* the refractive index of the medium ($n={\sqrt{\varepsilon }}_{r}$). The spontaneous emission rate can then be rewritten as $\Gamma_{r}\to n\Gamma_{0}$. The different refractive indices of the membranes considered in the current study amount to 1.378 for DOPC [73], 1.789 for DPPC (So) [74], and 1.555 for the 2:1 SM/Chol mixture [75]. For DPH the absorption and emission transition dipole moments for the first excited state are parallel to each other; the deviation with respect to the long axis of the molecule has been found to be less than 6.4°, confirming the nature of an effective cylindrically symmetric probe [76,77].

Based on the simulated emission intensities with parallel (*I_//_*) and perpendicular ($I_{⟂}$) polarizations, the fluorescence anisotropy of DPH can be computed as [78]:

(4) $$r=\frac{I_{//}-I_{⟂}}{I_{//}+2I_{⟂}}$$

Since this ratio depends on the orientations of the transition dipole moments, *r* expresses the influence of the biological environment on the depolarization of the fluorescence. To compute the parallel and perpendicular polarizations, use is made of the angle *b_ij_* between the initial transition dipole moment from state *j* to state *i* and the one at a time *t*, as [79]

(5) $$I_{∥}=\;\frac{4}{15\hbar^{4}c^{3}}\overset{j-1}{\underset{i=0}{\sum }}\omega_{ij}^{3}{\mu_{ij}}^{2}\left(1+2\cos^{2}b_{ij}\right)$$

(6) $$I_{⊥}=\;\frac{4}{15\hbar^{4}c^{3}}\sum_{i=0}^{j-1}\omega_{ij}^{3}{\mu_{ij}}^{2}\left(2-\cos^{2}b_{ij}\right)$$

For DPH, the fluorescence occurs from S_1_ to S_0_ and the analysis is restricted to these two states. 

## 4. Conclusions

The (non) linear optical and fluorescence properties of the conjugated probe diphenylhexatriene (DPH) were computed when embedded in three different membrane phases. Making use of hybrid quantum mechanics/molecular mechanics (QM/MM) methodologies and reverting to a set of uncorrelated snapshots selected from large scale molecular dynamics (MD) calculations, a localized one photon absorption spectrum is obtained for DPH embedded in both DOPC (Ld) and DPPC (So) membranes. On the other hand, when in SM/Chol the spectrum is broad, which can be rationalized by dihedral changes observed in the higher localized parts of the DPH molecule, while for a minor part of the snapshots a perpendicular orientation is seen with respect to the vertical z-axis of the membrane. DPH shows that the So phase of DPPC is the dominant one when TPA cross sections and hyper-Ryleigh scattering (HRS) are considered. Finally, the Lo phase of SM/Chol emerges when the decay of the fluorescence anisotropy is considered. This study proves that the combination of (non) linear optical and fluorescence methods can be profoundly used to recognize different membrane phases and paves the way for a general use of DPH as a probe to discriminate between them. We encourage experimentalists to test our findings and to use them in possible biomedical contexts in which aberrant cell membranes might be the indication for cancerous disorders. 

## Figures and Tables

**Figure A2:**
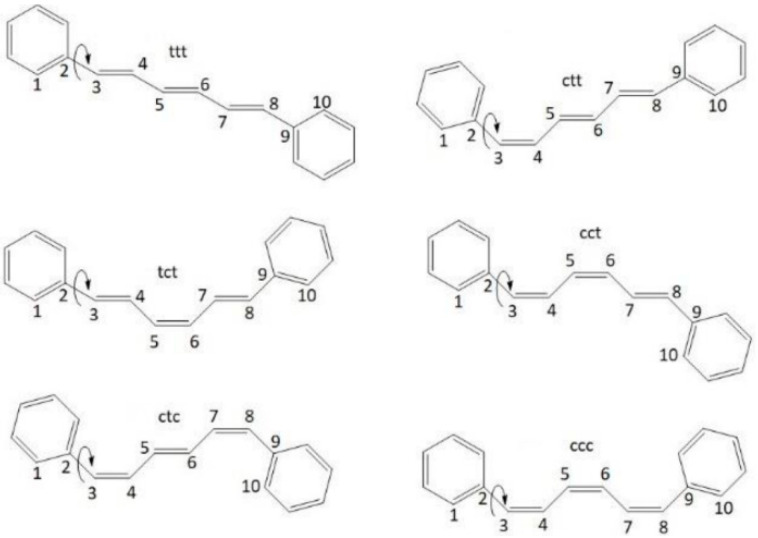
Different conformers of DPH.

**Figure A3:**
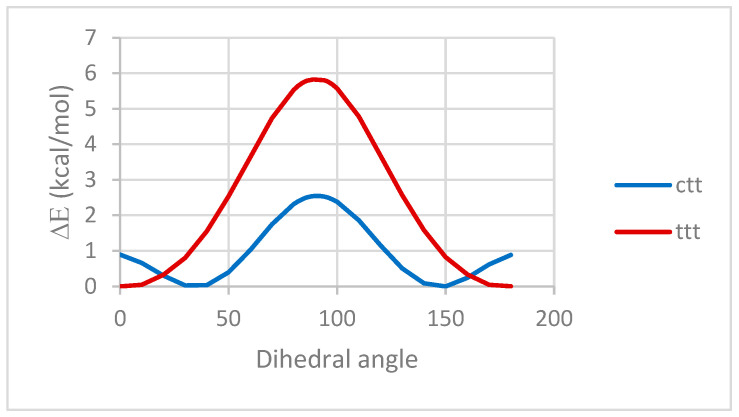
Ground state potential energy surface of the ctt and ttt conformers in which the dh1 dihedral angle (1-2-3-4, see Figure 1) is rotated from 0° to 180° while the rest of the backbone is kept constant (B3LYP/cc-pVDZ data). The trajectories of cct, ccc and ctc are identical to the one of ctt and are therefore not depicted by a color code.

**Figure A4:**
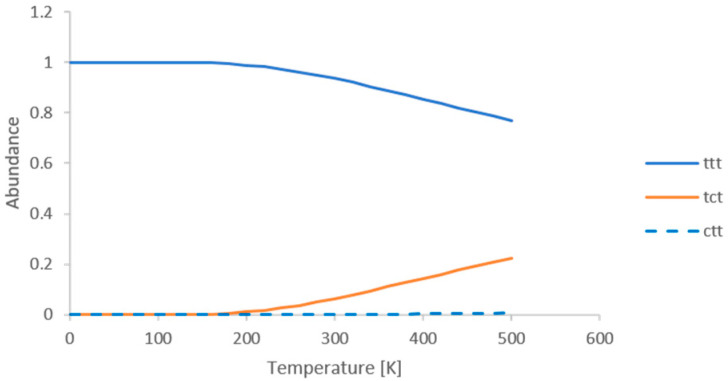
Conformational abundance of the ttt, tct and ctt conformers using a Boltzmann distribution in function of the temperature (B3LYP/cc-pVDZ data).

## Appendix A. Calculation of the Conformational Abundance

The rotational barriers over the single and double bonds of DPH are displayed in Figure A1. The plot was obtained by fixing a trans conformation while the respective dihedral angles are increased in steps. In (a) and (b), the rotations over the single bonds 2-3 and 4-5 are plotted, while in (c) and (d) the barriers over the double bonds 3-4 and 5-6 are given. Since the latter ones amount to more than 60 kcal/mol, the conformational flexibility of DPH in biological conditions can be attributed to the rotations over the four single bonds of the molecule.

However, it could be a choice of the spectroscopist to employ the conformers which differ from the all trans one by a rotation over the double bonds. In Figure A2, the different conformers are displayed and in Table A1 the energy differences with respect to the all trans one is given; the molecules are described by the “cis” (c, 30°) or “trans” (t, 180°) form of the dihedral angle over the respective double bond. Apart from the ttt conformer, the tct and ctt conformers are the least energetic ones with energy differences of 2.02 and 3.70 kcal/mol, respectively. The energy difference of more than 10 kcal/mol with the ccc conformer is the largest.

**Table A1:** Energy difference between the conformers of DPH (see Figure A1) *^a^.*

Conformer	ΔE
tct	2.02
ctt	3.70
cct	5.85
ctc	8.38
ccc	10.86

*^a^* Energy differences are calculated by means of B3LYP/cc-pVDZ and are given in kcal/mol relative to the ttt conformer. For the conformers ctt, cct, ccc and ctc, the optimized dihedral angle dh1 amounts to 150°; while for ttt and tct, the potential energy surfaces are found to be minimal at 0°.

As in the case of ttt, rotation barriers over the single bonds in the other conformers of Table A1 can be investigated, too. In Figure A3, the rotations over the dh1 dihedral angle for ttt and ctt are compared. The potential energy surfaces of cct, ccc and ctc are found to be identical to the one of ctt. The energy barriers at 90° for the latter ones amount to 2.5 kcal/mol, while for ttt it reaches 5.8 kcal/mol.

Making use of the energy differences, the conformational abundances can be calculated by means of a Boltzmann thermostatistical analysis, employing Gibbs’ free energy differences, thermal enthalpy and entropy corrections [37,80]. It can be noted that the B3LYP approach is known to provide harmonic vibrational frequencies of quality comparable to the coupled cluster level and thus to deliver sound partition functions in the analysis [81,82]. The results for a temperature window from 0 K to 500 K are given in Figure A4. It is seen that at ambient temperatures the abundance of the most important minor conformer does not exceed 6%. At a temperature as high as 470 K, 20% tct conformers might be found. The other conformers are even less important.

## Appendix B

A list of the linear absorption properties for DPH embedded in the three lipid membrane layers is given in Table A2. The S1 state is strongly allowed while the S2 and S3 are only weakly visible. For S3, the Λ-overlap parameter is the smallest, but is still larger than the critical value of 0.4, which would indicate a charge transfer character. Apart from minor contributions or rather limited changes, the S1 and S2 excited states are HOMO to LUMO and HOMO to LUMO+1 excitations, respectively. The HOMO-2 to LUMO transition contributes to the S3 for all three membranes. The depictions of the orbitals are given in Figure A6.

**Table A2:** Absorption properties for the first three excited states of the DPH probe embedded in the different membranes. Oscillator strength is reported in parentheses; all energy values are in nm *^a^.*

DOPC	Wavelength	Λ	Transition (%)
S1	336.8 (1.94)	0.83	H -> L (100 %)
S2	264.7 (0.01)	0.58	H -> L + 1 (90 %)
S3	257.0 (0.01)	0.46	H -> L + 2 (26 %)
			H − 2 -> L (23 %)
			H − 1 -> L + 3 (21 %)
**DPPC**	**Wavelength**	**Λ**	**Transition (%)**
S1	345.6 (1.91)	0.80	H -> L (100 %)
S2	276.5 (0.02)	0.56	H -> L + 1 (85 %)
S3	270.3 (0.01)	0.47	H − 2 -> L (35 %)
			H -> L + 3 (22 %)
**SMCHOL**	**Wavelength**	**Λ**	**Transition (%)**
S1	337.2 (1.65)	0.79	H -> L (100 %)
S2	256.1 (0.03)	0.55	H -> L + 1 (90 %)
S3	266.7 (0.02)	0.49	H − 1 -> L (35 %)
			H − 2 -> L (15 %)
			H − 1 -> L + 3 (15 %)

*^a^* H and L denote the highest occupied and lowest unoccupied molecular orbitals, respectively. Λ denotes the overlap parameter of Peach et al. [40].

## Appendix C

While for TPA (Table A3) and dynamic HRS (Table A4) the standard deviations (STD) are found in the expected range of 15–20%, the values obtained for the static HRS signal amount to a value as high as 75%. Since HRS is related to the orientation of the long axis of the probe, the STDs might be explained by the motions of the probes, which are hindered to a different extent in the different membrane phases [41]. In DPPC (So), the STD is the lowest among the different membranes, because the probe experiences a steric hindrance and a strongly limited rotation, while in the other two membranes these motions are less restrained, which explains the observed higher variation. The opposite is true for SM/Chol, for which the highest STDs are found. Furthermore, this is a manifestation of the conformational changes which were found for DPH in this membrane through the MD dihedral analysis reported in Section 2.1.

**Table A3:** Average values and standard deviations (STD) for the simulated TPA (two photon absorption) spectra (in GM units). The relative STD is given in parentheses.

TPA DOPC	Aver	STD (%)
S1	8.87	1.78 (20)
S2	131.3	33.62 (26)
S3	27.6	3.45 (12)
**TPA DPPC**	**Aver**	**STD (%)**
S1	61.1	8.94 (15)
S2	51.6	10.26 (20)
S3	48.8	11.74 (24)
**TPA SM/Chol**	**Aver**	**STD (%)**
S1	43.8	13.15 (30)
S2	51.6	9.49 (18)
S3	48.8	10.41 (21)

**Table A4:** Average values and standard deviations (STD) for the static and dynamic HRS signals (in 10^−30^ esu). The relative STD is given in parentheses.

HRS static.	Aver	STD (%)
DOPC	5.33	3.81 (70)
DPPC	17.7	5.85 (33)
SM/Chol	7.07	5.35 (75)
**HRS 810 nm**	**Aver**	**STD (%)**
DOPC	35.7	5.27 (15)
DPPC	128.7	18.5 (14)
SM/Chol	62.4	11.3 (18)
